# Validity and Reliability of New Equations for the Prediction of Maximal Oxygen Uptake in Male and Female Elite Adolescent Rowers

**DOI:** 10.2478/hukin-2022-0053

**Published:** 2022-09-08

**Authors:** Evgenia D. Cherouveim, Spyridon K. Methenitis, Theocharis Simeonidis, Panagiotis Georginis, Yiannis E. Tsekouras, Chrisa Biskitzi, Charis Tsolakis, Panagiotis Koulouvaris

**Affiliations:** 1 Sports Excellence, 1st Orthopedics Department, School of Health Sciences, National and Kapodistrian University of Athens, Athens, Greece; 2 School of Physical Education & Sports Science, National and Kapodistrian University of Athens, Athens, Greece; 3 Sports Performance Laboratory, School of Physical Education & Sports Science, National and Kapodistrian University of Athens, Athens, Greece; 4 Department of Nutrition and Dietetics, School of Health Science & Education, Harokopio University of Athens, Athens, Greece

**Keywords:** rowing performance, youth athletes, children, rowing test, maximal oxygen uptake

## Abstract

The aim of this study was to develop accurate, reliable, and reproductive equations for the prediction of maximum oxygen uptake ($\dot{V}$O_2max_) in male and female high-level adolescent rowers. This study included two parts. In the first part, V̇O_2max_ was evaluated in 106 male and 83 female high-level adolescent rowers during an incremental step test (IRT) on a rowing ergometer, and stepwise multiple regression analyses were used for the development of new equations. In the second part, these equations were tested in 26 new high-level adolescent rowers of the same age and anthropometrical characteristics (boys: 15.27 ± 2.70 yrs and 15.34 ± 2.80 yrs; 72.37 ± 10.96 kg and 70.96 ± 10.65 kg; girls: 15.00 ± 2.11 yrs and 15.94 ± 2.71 yrs; 62.50 ± 7.14 kg and 63.41 ± 6.72 kg for parts 1 and 2, respectively; p > 0.05). V̇O_2max_ was predicted from the combination of lean body mass (LBM) and the distance covered during the last 4 min stage of the IRT (boys: r^2^ = 0.715, F = 68.74, p = 0.001; girls: r^2^ = 0.769, F = 57.81, p = 0.001). In the second part, no significant differences were identified when the new equations were tested against measured V̇O_2max_ (boys: 3971.15 ± 713.38 mL·min^−1^ vs. 3915.83 ± 704.43 mL·min^−1^; girls: 3272.75 ± 551.46 mL·min^−1^ vs. 3308.94 ± 557.59 mL·min^−1^ for measured and predicted values, respectively; p > 0.05). In conclusion, V̇O_2max_ of high-level adolescent rowers can be predicted with high accuracy, reliability, and repeatability using simple and easily evaluated anthropometric and performance variables.

## Introduction

Rowing is a strength–endurance event in which performance depends on several physiological factors including rowers’ body composition, fiber type, functional aerobic and anaerobic capacity, mean and peak power output during rowing tests, and power output at 4 mmol·L^−1^ concentration of blood lactate (Ingham et al., 2002; Nevill et al., 2011). Among these factors, maximum oxygen uptake (*V̇*O*_2max_*) appears to be the most important factor of rowing performance, at least in moderate and well-trained adult rowers of both genders (Ingham et al., 2002; Nevill et al., 2011). Indeed, 65–80% of the energy demand for an all-out 2000 m rowing event is provided by aerobic energy pathways, while contribution of the anaerobic system varies between 12 and 35% (Ingham et al., 2002; Pripstein et al., 1999). Thus, increased *V̇O*_*2max*_ is associated with better 2000 m rowing performance, in both males and females, and also in elite and moderately trained rowers, regardless of their sports level (Ingham et al., 2002; Nevill et al., 2011; Pripstein et al., 1999). Indeed, it seems that regular evaluation of rowers’ *V̇O*_2*max*_ is needed because it can provide important data to sports scientists and coaches about physical fitness and performance status of their athletes and is also essential for the success of their training programs.

The evaluation of *V̇O*_2max_ under laboratory and field conditions using open-circuit automated gas analysis systems is still the most reliable and accurate procedure for the evaluation of *V̇O*_2*max*_. Unfortunately, it is not always possible to regularly perform this measurement because it requires expensive equipment and specialized personnel. However, it is essential for rowers to evaluate their *V̇O*_2*max*_ frequently during their training cycles (Klusiewicz et al., 2016). Thus, many research attempts have been made to develop reliable equations for the prediction of either rowers’ *V̇O*_2*max*_ or rowing performance using sub- or maximal exercise protocols and the physiological responses of rowers during these tests as well as rowers’ anthropometric characteristics (Akça, 2014; Klusiewicz et al., 2016; Klusiewicz and Faff, 2003; Otter et al., 2015). However, the majority of these studies provide equations to predict 2000 m performance for adult rowers using anthropometric variables or/and physiological responses, while only few studies have developed a reliable and reproductive equation for the prediction of rowers’ *V̇O*_2*max*_. In addition, there is a lack of reproducibility and reliability analyses of these equations in other rowers except from those used for the development of these equations. Verification of equations’ accuracy, reliability, and reproducibility in external populations and further statistical analyses (e.g., standard error of measurements, Bland & Altman 95% limits of agreements, standard error of the limits, inter-assay coefficient of variation) are needed (Atkinson and Nevill, 1998; Bland and Altman, 1986; Hopkins, 2000; Kottner et al., 2011). Finally, until recently, only two studies have reported reliable prediction equations for the determination of rowing performance in youth athletes (Mikulić and Ružić, 2008; Russell et al., 1998), while none have provided a reliable and reproducible equation for the prediction of adolescent rowers maximum oxygen uptake. Children and adolescents have significant differences in physiological characteristics and exercise/training responses compared to adult athletes (Engel et al., 2014; Zalavras et al., 2015). Therefore, the existing equations for the prediction of adult rowers’ *V̇O*_2*max*_ cannot be used in children and adolescents. Thus, the aim of this study was to develop accurate, reliable, and reproducible equations for the prediction of *V̇O*_2*max*_ in male and female high-level adolescent rowers based on anthropometric and performance characteristics, as well as physiological responses during an incremental indoor rowing step test.

## Methods

### Participants

A total of 215 (N = 215), 119 boys and 96 girls, members of a long-term athlete’s development (LTAD) program of the national rowing federation participated in this study. All participants were divided into four subgroups, as has been previously described (participants' characteristics are presented in Table 1). No significant differences were identified between the participants of the 1^st^ and 2^nd^ part of this study (*p* > 0.05; Table 1). All procedures were performed in accordance with the Declaration of Helsinki and approved by the local University ethics committee; a detailed written description of the procedures was sent to the parents, and written parental consent forms were obtained prior to the entry of each athlete in the training camp.

**Table 1:** Anthropometric characteristics and exercise capacity of participants.

	Participants of 1^st^ Part		Participants of 2^nd^ Part	
	Boys (N=106)	Girls (N=83)	Boys (N=13)	Girls (N=13)
Age (yrs)	15.27 ± 2.70	15.00 ± 2.11	15.34 ± 2.81	15.94 ± 2.71
Body Mass (kg)	72.37 ± 10.96	62.50 ± 7.14	70.96 ± 10.65	63.41 ± 6.72
Body Height (cm)	179.76 ± 7.92	167.93 ± 6.14	179.65 ± 7.61	167.39 ± 6.51
Body Fat Content (%)	16.03 ± 4.19	24.53 ± 3.94	15.32 ± 3.44	24.20 ± 2.65
Lean Body Mass (kg)	57.04 ± 10.23	45.62 ±5.07	58.81 ± 10.20	46.82 ± 4.77
Training Experience (yrs)	5.02 ± 2.20	5.15 ± 1.93	4.89 ± 3.02	5.01 ± 2.90
2000(m:s) m Rowing Performance	6:62 ± 0:45	7:48 ± 0:51	6:45 ± 0:62	7:56 ± 0:66
Maximum (mL·min^-1^) Oxygen Uptake	3991.74 ± 345.99	3058.10 ± 169.95	3971.15 ± 713.38	3272.75 ± 551.46
Maximum Body Mass Oxygen (mL·kg^-^Uptake ^1^·min^-1^) per	55.74 ± 7.97	49.12 ± 6.11	54.99 ± 8.12	50.02 ± 5.01
Distance Last 4 min Covered Trial (m) During the	1175.78 ± 102.12	1021.73 ± 100.80	1155.85 ± 121.24	1034.38 ± 66.21
Average Last 4 min Stroke Trial Rate (spm) During the	32.54 ± 1.92	33.62 ± 2.67	34.04 ± 2.01	30.99 ± 2.89
Mean Last 4 Power min Trial Output (W) During the	330.72 ± 74.01	223.71 ± 41.85	324.38 ± 88.50	228.75 ± 45.15
Maximum Heart Rate (bpm)	188.39 ± 12.39	187.89 ± 11.95	187.88 ± 10.73	185.62 ± 17.62

*Values are mean ± SD. No significant differences were found for the comparison between boys and girls from the first and second parts of the present study (p > 0.05)*.

### Experimental Approach for the Problem

This study included two parts. The first part was used to develop new equations for the prediction of *V̇O*_2*max*_ in adolescent well-trained rowers based on easily evaluated variables, without the need of any expensive and/or sophisticated equipment. The second part was used as a validation study for the determination of reliability and reproducibility of the new equation in a different group of youth well-trained rowers. Participants in both parts of the study were members of a high-level national rowing group, in which, each athlete was selected from the national rowing federation to participate in an LTAD program based on his/her results in national and international rowing competitions. This study was performed during the first 3 days of a training camp, organized by the national rowing federation, four weeks after the under 18 yrs national championship. The inclusion criteria were: 1) age range between 13 and 17 years old, 2) absence of restraining orthopedic/neuromuscular maladies, 3) weight stability (±2 kg) prior to entry (~1 month), and 4) absence of medications that are known to affect rowing performance. Participants who fulfilled the inclusion criteria were randomly allocated to one of the four different groups (2 main and 2 verification groups) based on their gender and their body composition variables (lean body mass and body fat content), using an MS Excel algorithm. Specifically, this algorithm chose in random order from the initial 119 boys and 96 girls, 13 boys and 13 girls, allocated them in two new subgroups (one per gender), and tested whether there were any significant differences in the mean values of lean body mass and body fat content between the main and new subgroups of boys and girls, separately. If a difference was observed, then the algorithm chose other 13 boys and/or 13 girls, until no significant differences in the abovementioned variables between the main and verification groups of boys and girls were observed. Thus, 106 boys and 83 girls were allocated into two subgroups for the development of new equations (1^st^ part) for male and female rowers separately, while the remaining 13 boys and 13 girls were assigned into the verification subgroups (2^nd^ part). All participants reported to the laboratory (air temperature 24–26°C and humidity 40–45%) between 08:00 and 12:00 am during their evaluation day, after at least 3-day rest, and had their anthropometric and body composition evaluations. Then, they performed an incremental test to exhaustion on a rowing ergometer (Ingham et al., 2013) for the determination of *V̇O*_2*max*_ after a standard 10 min warm-up.

### Design and Procedures

#### Evaluation of body composition and anthropometric characteristics

Body height was measured using a stadiometer with accuracy of 0.5 cm (SECA 220, Seca Corporation, Columbia, USA). Body mass was evaluated using a calibrated digital scale with accuracy of ±100 g (Seca 707, Seca Corporation, Columbia, USA). Body composition was estimated using the skinfold thickness method developed by Jackson and Pollock (1985). Skinfold measurements were obtained from nine sites, i.e., at the bicipital, tricipital, subscapular, suprailiac, abdominal, midaxillary, pectoral, anterior thigh levels, and calf using the Lange caliper. A minimum of two measurements were made at each skinfold site by the same highly experienced investigator. Fat-free body mass (FFM) was calculated as the difference between body mass and body fat, and the sum of eight skinfolds was calculated. Participants were instructed to remove shoes and unnecessary clothing. The intraclass correlation coefficient (ICC) for body fat was 0.93, (95% CI: Lower = 0.89, Upper = 0.97), and for LBM 0.98, (95% CI: Lower = 0.95, Upper = 0.99), (*p* < 0.0001, n = 10) (Papadopoulou et al., 2018).

#### Evaluation of V̇*O*_2*max*_

For the determination of participants’ *V̇O*_2*max*_, an incremental step test, composed of 5 × 4 min stages, was adapted, according to previous research (Ingham et al., 2013), after a 10 min self-paced incremental warm up. All evaluations were performed on an air-braked rowing ergometer (Concept II C, Nottingham, UK). This test was selected because it provides a very reliable estimation of 2000 m rowing performance (Ingham et al., 2013) and also because all participants were familiar with this test from previous performance evaluations. Drag factors were 140 for boys and 130 for girls. The intensity between stages 1–4 was increased by 25 W and 2 strokes/min, followed by 30 s of rest. Between stages 4 and 5, a 150 s rest interval was allowed. Then, participants performed the last stage, with a maximum self-paced effort (Ingham et al., 2013). The screen of an ergometer was set to display the remaining time, average 500 m distance, pace rate/500 m, and accumulated distance. Verbal encouragement was given during the last minute of the test. The distance covered, average stroke rate, and mean power output during the 4 min trial were evaluated. The heart rate was continuously monitored by telemetry (Sport tester ^TM^ Polar, Kempele, Finland). During the test, gas exchange and ventilatory variables were continuously recorded breath by breath using a portable open-circuit automated gas analyzer system (K5, COSMED, Italy). Calibration was performed before each test using a 3-l calibration syringe with two different gas mixtures. *V̇O*_2*max*_ was considered the highest mean value recorded during the last 10 s of the 4 min all-out rowing test, as has been previously suggested (Martin-Rincon et al., 2019) when at least two of the following criteria were met: a) the heart rate within 10% of age-predicted maximum, b) the respiratory exchange ratio ≥1.15, and c) blood lactate concentration greater than 8–9 mmol/L. Two independent investigators analyzed the plots for the determination of *V̇O*_2*max*_ for each athlete. The ICC for *V̇O*_2*max*_ was 0.87 (95% CI: Lower = 0.81, Upper = 0.94; n = 7).

### Statistical Analysis

Shapiro–Wilk and Kolmogorov–Smirnov tests were used to assess the normality of data. No violations of normality distribution were identified (*p* > 0.05). All data are presented as the mean and standard deviation (± SD). Multiple regression analyses (stepwise) were performed to evaluate the best linear combination for prediction of *V̇O*_2*max*_ in boys and girls separately, based on multiple regression analysis assumptions and the results of Pearson’s product moment correlation coefficient analyses, which were used to determine the relationships between the variables (data are not presented). According to multiple regression analysis assumptions and the results of Pearson’s r correlations, the following variables were entered in multiple regression analyses: lean body mass, covered distance and mean power output during the last 4 min of the trial, maximum heart rate, and average stroke rate during the last 4 min of the trial.

Independent samples Kolmogorov–Smirnov test (K.S. test) was employed; it assumed as a null-hypothesis that the distributions of predicted and measured *V̇O*_2*max*_ were equal. In addition, as previously suggested (Atkinson and Nevill, 1998; Bland and Altman, 1986; Hopkins, 2000; Kottner et al., 2011), for the determination of reliability, agreement, and reproducibility between the predicted and measured *V̇O*_2*max*_, the following analyses were performed: intraclass correlation coefficient (ICC; two factor mixed effects model; consistency type), standard error of measurements $\left(\text{SEM}=SD\cdot \;\sqrt{1-ICC}\right),$inter-assay coefficient of variation

$\left(\text{CV}=\sqrt{Mean\;Square\;Error\cdot Grand\;mean^{-1}}\right.$100), Bland & Altman 95% limits of agreements (LOA), standard error of the limits $\left(\text{SEL}=\sqrt{3\cdot Standrad\;Deviation\;of\;Difference^{2}}\cdot n^{-1}\right),$95% confidence interval for the limits of agreement (CILOA = 95% LOA ± (1.96 ∙ *SEL*), and repeatability coefficient (e.g., the maximum difference that is likely to occur between repeated measurements;


$$RC=1.96\sqrt{\left.2\cdot StandradDeviationofDifference^{2}\right)}.$$

ICCs values between 0.800 and 1, SEMs and means of the differences near zero, CVs < 10%, as well as low values/ranges at LOAs, SELs, and RCs analyses are thought to be indicators of the absolute reliability agreement and reproducibility of the measurements (Atkinson and Nevill, 1998; Bland and Altman, 1986). Statistical analysis was performed with the SPSS Statistics Ver. 20 (IBM Corporation, USA). Statistical significance was accepted at *p* ≤ 0.05 for all tests.

## Results

### Results from the first part of the study

Anthropometric and performance characteristics are shown in Table 1. Multiple regression analyses revealed two models for the prediction of *V̇O*_2*max*_ in boys and girls. The equations are as follows:

**(1) Equation for the estimation of *V̇O*_2*max*_ in boys**:

***V̇O***_**2*****max***_ **(mL·min−1)**: −2310.815 + (Lean Body Mass · 40.991) + (Covered Distance During the Last 4 min Trial · 3.365) [r = 0.863, r^2^ = 0.715, F = 68.74, *p* = 0.000]

**(2) Equation for the estimation of *V̇O***_**2*****max***_ **in girls**:

***V̇O***_**2*****max***_ **(mL·min−1)**: −572.696 + (Lean Body Mass · 41.182) + (Covered Distance During the Last 4 min Trial · 1.707) [r = 0.877, r^2^ = 0.769, F = 57.81, *p* = 0.000]

When comparing the distributions of estimated and measured *V̇O*_2*max*_ values, K.S. tests revealed no significant differences (*p* values: boys = 0.975, girls = 0.982). Table 2 shows reliability statistics between the predicted and measured *V̇O*_2*max*_ values for both boys and girls. ICCs were over 0.908 (*p* = 0.000); CVs ranged between 5.78 and 6.41%; the mean of differences was −27.26 ± 56.58 mL·min^−1^ and −25.52 ± 64.32 mL·min^−1^ for girls and boys, respectively; low/small values and/or small range of LOA, SEL, and RC values were observed, which indicated the absolute reliability and reproducibility (Atkinson and Nevill, 1998; Bland and Altman, 1986) of the measurements (Table 2).

**Table 2:** Intra-rater reliability between predicted and measured maximum oxygen uptakes in rowers recruited to develop the new prediction equations.

		Boys (N=106)		Girls (N=83)	
		
		Measured	Estimated	Measured	Estimated
Mean ± SD (mL·min^-1^)		3834.61 ± 345.99	3991.75 ± 309.89	3058.10 ± 369.95	3034.01 ± 326.19
Grand Mean ± SD (mL·min^-1^)		3913.18 ± 639.23		3046.06 ± 347.40	
ICC	Anova (F; *p* < 0.05)	1.30		1.86	
	R (*p* = 0.000)	0.908		0.912	
	95% CI (Low-High)	0.846 – 0.925		0.871 – 0.949	
SEM (mL·min^-1^)		193.88		103.24	
CV (%)		5.78		6.41	
LOA (mL·min^-1^)	MeanDiff ± SD_Diff_	-27.26 ± 56.58		-25.52 ± 64.32	
	95% CI_Diff_	-38.16 to -16.36		-28.43 to -0.34	
	High CILOA) 95% of LOA (95%	83.63 (81.824 – 85.44)		111.67 (109.04 – 114.30)	
	Low CILOA) 95% of LOA (95%	-138.15 (-139.96 - -136.34)		-140.46 (-143.30 - -137.83)	
	LOA 95% Width	221.79		252.13	
	SEL	0.924		1.34	
	RC	156.83		178.28	

*ICC: Intraclass correlation coefficient; SEM: standard error of measurement; CV: inter-assay coefficient of variation; LOA: Bland & Altman 95% limits of agreements; MeanDiff: mean of the difference between measured and predicted values; SDDiff: standard deviation of the difference between measured and predicted values; CI: confidence interval; CI_Diff_: confidence interval of the difference between measured and predicted values; 95% CILOA: 95% confidence interval for the limits of agreement; SEL: standard error of limits; RC: repeatability coefficient*.

### Results from the second part of the study

For the evaluation of accuracy, reliability, and reproducibility of the new equations, we tested them against two independent subgroups of boys and girls with the same characteristics as those in the first part of this study (no significant differences were identified between the subgroups of boys or girls from the two parts of this study; *p* > 0.05; Table 1). Reliability statistics between the predicted and measured *V̇O*_2*max*_ values, for the second part of the study, are shown in Table 3. K.S tests revealed no differences between the distributions of estimated and measured *V̇O*_2*max*_ values (*p* values: boys = 0.998, girls = 0.998). ICCs, CVs, mean of differences of LOA, SEL, and RC values revealed high reliability and reproducibility of the new equations, even when they were tested against two new subgroups of adolescent rowers who were not included in the analyses for the creation of these two equations (Table 3).

**Table 3:** Intra-rater reliability between predicted and measured maximum oxygen uptake in 26 rowers recruited for the evaluation of the new prediction equations.

		Boys (N=13)		Girls (N=13)	
	
		Measured	Estimated	Measured	Estimated
Mean ± SD (mL·min^-1^)		3971.15 ± 713.38	3915.83 ± 704.43	3272.75 ± 551.46	3308.94 ± 557.59
Grand Mean ± SD (mL·min^-1^)		3943.49 ± 709.47		3223.84 ± 600.16	
ICC	Anova (F; *p* < 0.05)	2.650		2.520	
	R (*p* = 0.000)	0.980		0.985	
	95% CI (Low-High)	0.900 – 0.991		0.973 – 0.993	
SEM (mL·min^-1^)		100.33		73.50	
CV (%)		3.33		2.07	
LOA (mL·min^-1^)	MeanDiff ± SD_Diff_	-29.12 ±63.84		-24.42 ± 53.61	
	95% CI_Diff_	-54.27 to -3.96		-57.77 to 8.93	
	High CILOA) 95% of LOA (95%	86.17 (64.33– 97.68)		80.65 (66.65 – 94.65)	
	Low CILOA) 95% of LOA (95%	-174.25 (-185.93 - -152.58)		-129.49 (-143.49 - -115.49)	
	LOA 95% Width	250.26		210.15	
	SEL	8.50		7.14	
	RC	176.96		148.59	

*ICC: Intraclass correlation coefficient; SEM: standard error of measurement; CV: inter-assay coefficient of variation; LOA: Bland & Altman 95% limits of agreements; MeanDiff: mean of the difference between measured and predicted values; SDDiff: standard deviation of the difference between measured and predicted values; CI: confidence interval; CIDiff: confidence interval of the difference between measured and predicted values; 95% CILOA: 95% confidence interval for the limits of agreement; SEL: standard error of limits; RC: repeatability coefficient*.

## Discussion

The main result of this study was that male and female adolescent rower’s *V̇O*_2*max*_ could be predicted by the linear combination of lean body mass and the distance covered during the last 4 min stage of the incremental step rowing test, with significant accuracy. In addition, compared to previous reports, two important methodological differences of this study are that: firstly, this study adapts all needed statistical analyses, which are crucial in this type of study, for the investigation of absolute accuracy, reliability, and reproducibility of the developed equations against the actual measured *V̇O*_2*max*_ values of male and female adolescent rowers; secondly, the new equations were tested in external populations. Unfortunately, until now, the majority of studies in this field focused mainly on ICC values. However, the determination of the ICC only cannot provide sufficient evidence about the accuracy, reliability, and reproducibility between the actual measured and predicted values (Atkinson and Nevill, 1998; Bland and Altman, 1986; Hopkins, 2000; Kottner et al., 2011).

**Figure 1:**
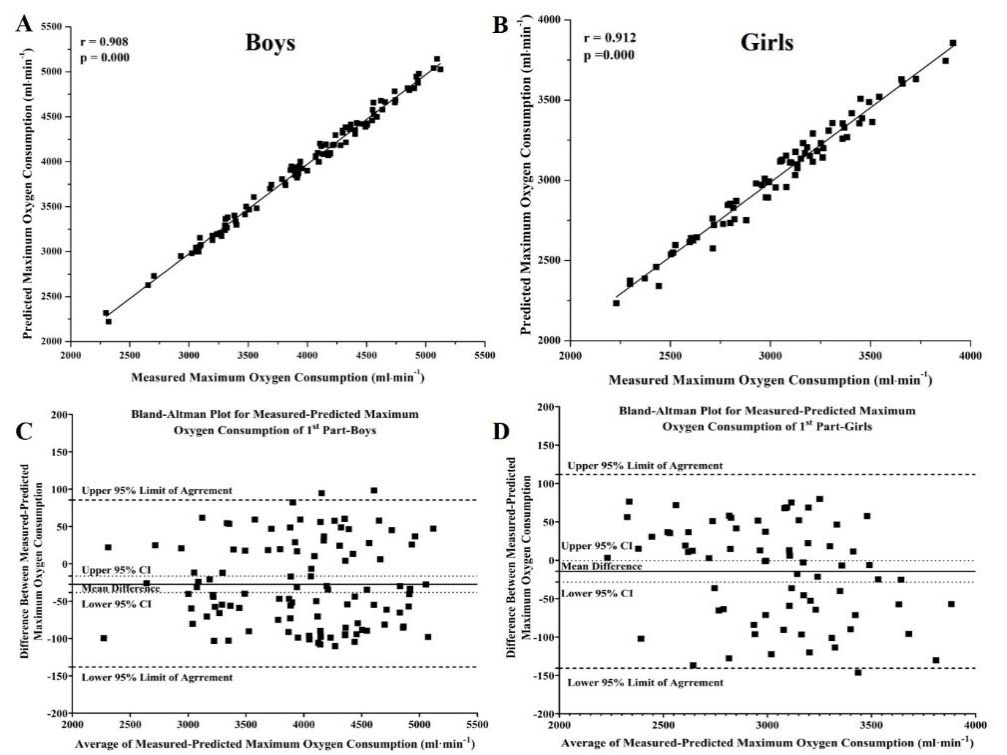
Correlation plots (A & B) and Bland-Altman 95% limits of agreements plots (D & C) for the comparisons between measured and predicted maximum oxygen uptake for boys (N = 106; A & C) and girls (N = 83; B & D) of the present study’s first part, respectively.

**Figure 2:**
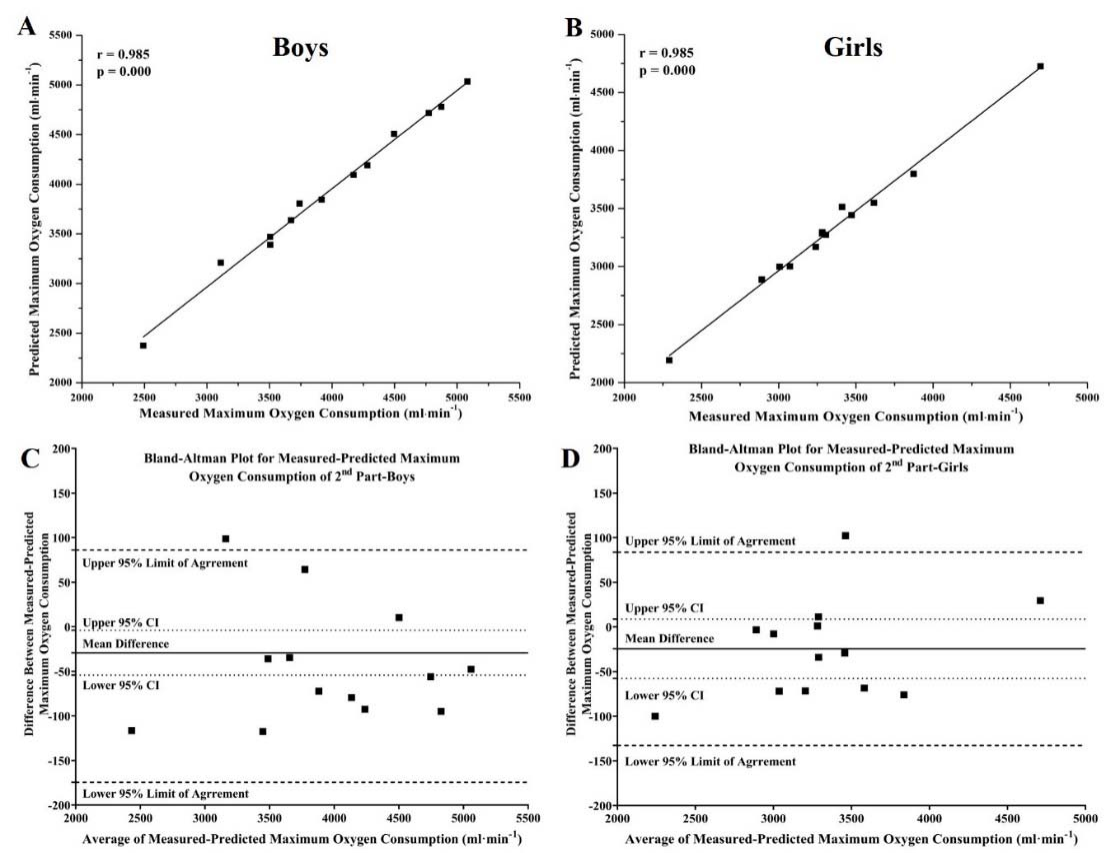
Correlation plots (A & B) and Bland-Altman 95% limits of agreements plots (D & C) for the comparisons between measured and predicted maximum oxygen uptake for boys (N = 13; A & C) and girls (N = 13; B & D) of the present study’s second part, respectively.

Thus, in studies aiming to develop and provide new equations for the estimation of a biological variable or performance in elite athletes or patients, further statistics are needed such as SEM, CV, mean of the differences, Bland–Altman plots, LOA, SEL, and RC, to inform readers and professionals about the extent of error existing in any diagnosis using the new developed equations, as has been previously suggested (Atkinson and Nevill, 1998; Bland and Altman, 1986; Hopkins, 2000; Kottner et al., 2011). According to the results of these analyses, in this study, all established criteria for high accuracy, reliability, repeatability, and reproducibility have been met, which indicates that these new equations for the prediction of *V̇O*_2*max*_ of male and female adolescent rowers can be used by sports scientists and coaches, which will provide them with data that are identical to those that they would receive during direct evaluation of *V̇O*_2*max*_ in laboratory and field settings using open-circuit automated gas analyzers. In addition, high values of accuracy, reliability, repeatability, and reproducibility have been found when new equations were tested in two external groups (participants of these groups were not used for the development of these equations) of adolescent well-trained rowers with the same gender, age, and rowing performance, which further supports the usefulness of these new equations for the prediction of adolescent rowers’ *V̇O*_2*max*_. Unfortunately, previous studies do not provide data either on young or adult rowers that can be used to compare the results of accuracy, reliability, repeatability, and reproducibility analyses in this study.

In both equations, lean body mass seems to be the factor with the highest predictive value for adolescent rowers’ *V̇O*_2*max*_. Indeed, body mass, but mostly lean body mass and muscularity, have been repeatedly reported to be highly correlated with rowing performance and rowers’ *V̇O*_2*max*_ (Akça, 2014; Ingham et al., 2002; Klusiewicz et al., 2016; Otter et al., 2015). Rowing is an aerobic-type exercise, which demands the activation of almost every muscle of the human body (Secher et al., 1982); increased rower muscularity leads to greater peak power output during a rowing test (Akça, 2014; Klusiewicz and Faff, 2003; Otter et al., 2015; Yoshiga and Higuchi, 2003). In addition, in both adults and children, increased lean body mass has been linked to increased capacity of O_2_ blood extraction and stroke volumes, cardiovascular function, the number and density of capillary and mitochondria content per muscle fiber, oxidative capacity, larger vascular bed, and muscle pumps facilitating greater venous return, which leads to higher values of *V̇O*_2*max*_ (AlKandari and Nieto, 2019; Carrick-Ranson et al., 2012; Drarnitsyn et al., 2009; Egan and Zierath, 2013; Lolli et al., 2017). Thus, it was expected that lean body mass of adolescent rowers should be one of the most important variables determining their *V̇O*_2*max*_, as has been previously reported in adult rowers (Akça, 2014; Drarnitsyn et al., 2009; Ingham et al., 2002; Nevill et al., 2011).

The distance covered during the last four min trial of the incremental step test seems to be the next most significant variable determining *V̇O*_2*max*_. The greater lean body mass and distance covered suggest rowers’ ability to produce and maintain higher levels of muscle power for longer periods, most likely because these rowers have greater metabolic and movement efficiency and, thus, rowing economy (Cosgrove et al., 1999; Ingham et al., 2002; Pripstein et al., 1999; Secher et al., 1982). As previously reported, rowing is an endurance–strength sport, requiring increased ability for energy production from both aerobic and anaerobic energy systems (Ingham et al., 2002; Pripstein et al., 1999). Indeed, increased values of both aerobic and anaerobic capacities/power have been linked to better rowing performance, which indicates that the ability of a rower to produce and maintain an increased amount of muscle forces/power over a longer period directly affects his/her rowing performance (Akça, 2014; Ingham et al., 2002; Nevill et al., 2011; Secher et al., 1982). Thus, in this study, the inclusion of this variable in the new developed equations for the prediction of *V̇O*_2*max*_ in either male or female adolescent rowers seems expected and logical.

## Conclusions

In conclusion, the data obtained in this study suggest that *V̇O*_2*max*_ of male and female adolescent rowers can be predicted with high accuracy, reliability, repeatability, and reproducibility using simple and easily evaluated anthropometric and performance variables during an incremental indoor rowing step test, without the use of any expensive and/or sophisticated equipment or without the need for specialized personnel. The results of this study have significant practical implications. They allow sports scientists and coaches of elite youth rowers to assess *V̇O*_2*max*_ of their athletes anytime and everywhere in an effort to frequently evaluate their physical fitness and performance status and also to evaluate the progress of their training programs.
